# The dose-adherence model: a critical review of the computation of level of adherence to antiretroviral drugs

**DOI:** 10.1186/1742-4690-8-S2-P57

**Published:** 2011-10-03

**Authors:** D Ofosu, P Agyei-Baffour, G Acquah-Haqan, AJ Amankwa, ENL Browne

**Affiliations:** 1 Komfo Anokye Teaching Hospital, Kumasi, Ghana; 2 Department of Community Health, School of Medical Sciences, College of Health Sciences, Kwame Nkrumah University of Science and Technology, Kumasi, Ghana

## Objectives

To develop a model for determining the level of dose adherence of Antiretroviral Therapy (ART) clients from the first day of ART to the day of interview and to determine the dose adherence levels of the ART clients using the model.

## Methods

A cross-sectional study was conducted using pre-tested standardized questionnaires in exitinterviews in three ART centres and eleven social support groups to determine the dose adherence levels of ART clients in the Eastern Region of Ghana. A dose-adherence model was developed from three types of dose records; the observed doses, the expected doses and the missed doses and the frequency at which clients defaulted since commencement of ART. This model encapsulated the short-term recall of missed doses and the long-term default frequency of ART clients to arrive at the observed / expected adherence level ratio; the expected doses being the theoretical number of doses to be taken from the first day of ART to the time of interview and the observed doses being the difference between the expected doses and the missed doses.

## Results

Standard adherence levels derived from the model based on literature were 25% [1], 50% [2], 80% [3] and 100% [4]. However, 83.2% of respondents were 100% adherent having honoured all their re-fill appointments and never missed doses since commencement of ART. 14% and 1.7% of the 725 respondents were 80% and 50% adherent respectively whereas the remaining 1.1 % were 25% adherent. Observed adherence levels were statistically significant at a p-value of 0.0517. Further analyses on factors affecting dose adherence were conducted on the respondent's knowledge, attitudes and practices, socio-demographic features, social support received, ARV dispensing consistency and sources of ART information and there was no significance over the adherence levels. Contrarily, the mechanisms adopted for adherence significantly (p-value = 0.0517) affected the adherence levels.

## Conclusion

The dose-adherence model complements other methods for deducing client's level of dose adherence by considering the long-term default frequency as an addition to the short-term recall of missed doses or self-report.
